# Subcellular localization of glypican-5 is associated with dynamic motility of the human mesenchymal stem cell line U3DT

**DOI:** 10.1371/journal.pone.0226538

**Published:** 2021-02-19

**Authors:** Masao Takeuchi, Kikuko Takeuchi, Tomoyo Takai, Ritsuko Yamaguchi, Tetsushi Furukawa, Ken-ichi Akagi, Jun K. Takeuchi

**Affiliations:** 1 Section of Laboratory Equipment, National Institutes of Biomedical Innovation, Health and Nutrition, Ibaraki-city, Osaka, Japan; 2 Division of Bio-informational Pharmacology, Medical Research Institute, Tokyo Medical and Dental University, Bunkyo, Tokyo, Japan; Michigan Technological University, UNITED STATES

## Abstract

Glypican-5 (GPC5) is a heparan sulfate proteoglycan (HSPG) localized to the plasma membrane. We previously reported that in the human mesenchymal stem cell line UE6E7T-3, GPC5 is overexpressed in association with transformation and promotes cell proliferation by acting as a co-receptor for Sonic hedgehog signaling. In this study, we found using immunofluorescence microscopy that in transformed cells (U3DT), GPC5 localized not only at primary cilia on the cell surface, but also at the leading edge of migrating cells, at the intercellular bridge and blebs during cytokinesis, and in extracellular vesicles. In each subcellular region, GPC5 colocalized with fibroblast growth factor receptor (FGFR) and the small GTPases Rab11 and ARF6, indicating that GPC5 is delivered to these regions by Rab11-associated recycling endosomes. These colocalizations suggest that GPC5 plays an important role in FGF2 stimulation of cell migration, which was abrogated by knockdown of GPC5. Our findings indicate that GPC5 plays a role in regulation of U3DT cell migration and provides several insights into the functions of GPC5 that could be elucidated by future studies.

## Introduction

Glypicans (GPCs) and syndecans (SDCs), which are heparan sulfate proteoglycans (HSPGs) displayed on the surface of most mammalian cells, have long been thought to act as co-receptors for cell-surface receptors in several signaling pathways, including Hedgehog (Hh), Wnt, bone morphogenetic protein (BMP), and fibroblast growth factor (FGF) signaling [1]. The glypican family includes six members (GPC1 to GPC6), each of which is linked to the plasma membrane through a glycosylphosphatidylinositol (GPI) anchor, whereas the syndecan family includes four transmembrane proteins (SDC1 to SDC4) [2]. Recently, we reported that Glypican-5 (GPC5) is dramatically overexpressed in association with transformation after prolonged culture of the human mesenchymal stem cell line UE6E7T-3, and that knockdown of GPC5 expression decreases cell proliferation [3]. GPC5 is overexpressed in rhabdomyosarcomas (RMS), and down-regulation of GPC5 expression by RNAi decreases the proliferation rate of RMS cells [4]. Subsequent work showed that GPC5 stimulates RMS cell proliferation by activating Hh signaling by promoting the binding of the ligand Sonic hedgehog (Shh) to Patched (Ptc), the Hh receptor on the cell surface [5]. Similar evidence has also been obtained in cerebellar granule cell precursors [6] and salivary adenoid carcinoma [7]. Conversely, overexpression of GPC5 inhibits prostate [8] and lung cancer [9] cell proliferation. In non-small cell lung cancer, some reports have suggested that GPC5 is a tumor promoter [10], whereas others insist that it is a tumor suppressor [11, 12].

Cell-surface HSPGs also function as potent co-receptors for FGF signaling, as well as Hh signaling; SDCs and GPCs modulate FGF activity by promoting binding of FGF to its receptors (FGFRs) [13]. In particular, they play roles in tumorigenesis and cancer progression. GPC1 is overexpressed in human pancreatic cancer cells [14], breast cancer cells [15], and gliomas [16], and it increases the proliferative response to FGF2, heparin-binding epidermal growth factor-like growth factor (HBEGF), and HGF. In addition, knockdown of HSPGs in these cancer cells decreases the rate of proliferation, suggesting that GPC1 potentiates FGF signaling. Likewise, GPC5 induces a greater increase in the proliferation rate of a RMS cell line in the presence of FGF2 [4]. However, GPC5 localizes near primary cilia in RMS cells [5] and neural precursors [6], indicating that GPC5 interacts with Ptc1 receptor in Hh signaling but not in FGF signaling. We also detected strong staining of GPC5 in the same perinuclear region as concentrated Ptc1 in immunostained U3DT cells [3]. Although these studies clearly establish HSPG as a co-receptor for FGF- or Hh-mediated signaling, the study of SDC4 signaling demonstrates that full activity of FGFs requires not only receptor interaction, but also internalization via HSPG-dependent pathways [17]. In addition, a recent study revealed a role for SDCs in vesicular trafficking and endocytic control during FGF signaling processes [18].

Although we demonstrated that GPC5 participates in U3DT cell proliferation, HSPGs have numerous cellular functions related to modulation of the Hh, Wnt, BMP, and FGF signaling pathways, depending on cell and tissue type. However, most studies of cell-surface HSPGs to date have focused on SDCs, and except for a few examples, less is known about GPCs, particularly GPC5. Therefore, in addition to its role as a co-receptor for Hh signaling, GPC5 might have diverse yet heretofore undescribed functions. As a first approach to identifying other GPC5 functions, we investigated the subcellular distributions of GPC5 during various processes in U3DT cells. We reasoned that an understanding of when and where GPC5 localizes within the cell might provide hints about other GPC5 functions.

Here, we show that GPC5 on the cell surface is associated with FGFR1 and small GTPases (Rab11 and ARF6) during cellular migration, and localizes at the leading edge of migrating cells, at the midbody area and plasma membrane blebs during late cytokinesis. In addition, we found many vesicles containing GPC5 in U3DT cell-culture medium. These localizations suggest that GPC5 plays a role in regulation of U3DT cell migration.

## Materials and methods

### Cell culture

**Fig 1:**
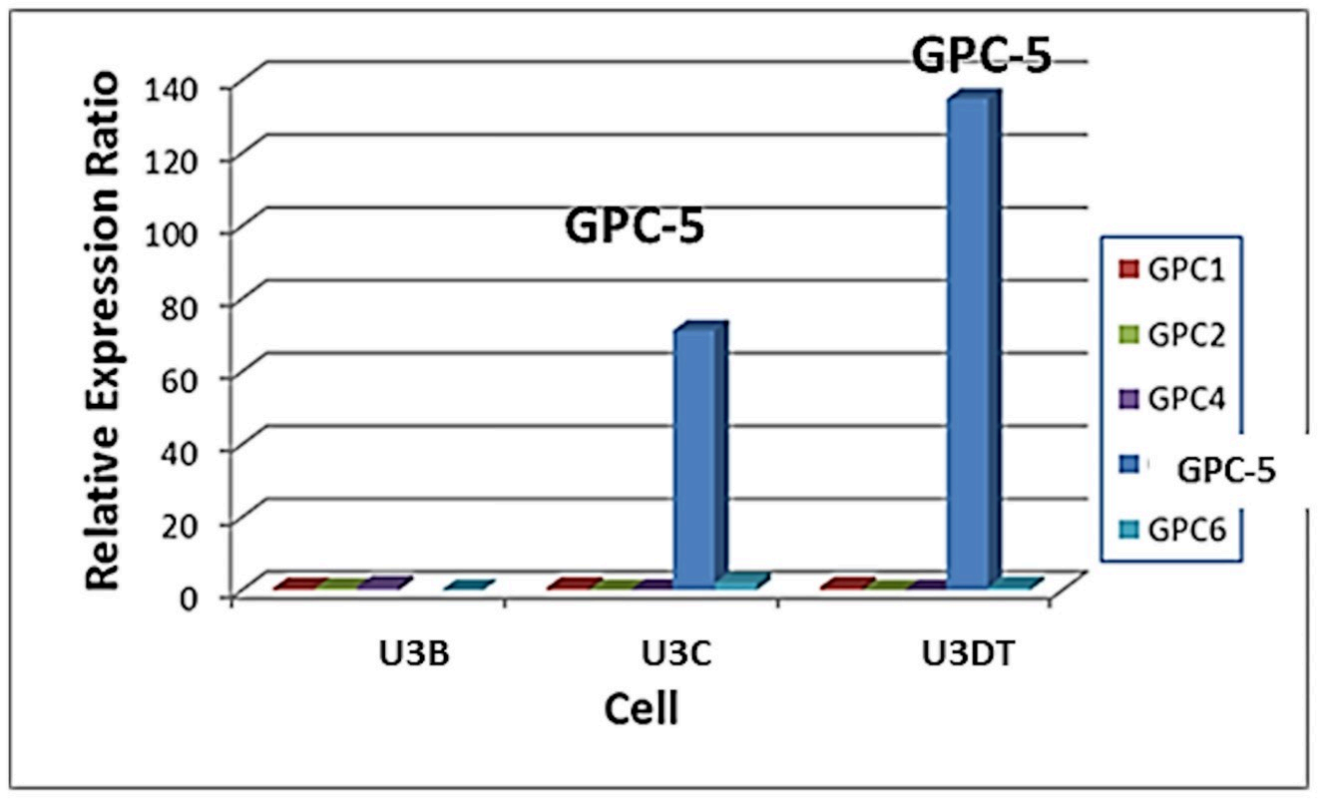
Alterations in GPC5 expression during long-term culture. Gene expression of GPCs (GPC1 to GPC6) at four culture stages were analyzed using data in the DDBJ database of the National Institute of Genetics (http://www.ddbj.nig.ac.jp/), accession number: DRA000533 [3]. Each value is shown relative to the corresponding value in U3-A cells (culture stage 1). GPC1, red; GPC2, green; GPC3, no expression; GPC4, purple; GPC5, blue; GPC6, light blue. As shown in the previous report [3], PDLs of U3-A, U3-B, U3-C and U3DT cells are 60‒90, 91‒150, 151‒230 and 231‒295, respectively.

The human mesenchymal stem cell line UE6E7T-3 (JCRB1136), which is the same as U3-A in Fig 1, and their transformed derivative U3DT (JCRB1136.01) were cultured in DMEM containing 10% FBS as described in a previous report [3]. In this paper UE6E7T-3 and U3DT cells cultured below ten passages were used for following experiments.

### Preparation of extracellular vesicles (EVs)

EVs were prepared from conditioned medium of U3DT cells cultured for 2 days in DMEM with 0.1% FBS, which had previously been centrifuged at 105,000 *g* for 90 min. The conditioned medium was centrifuged at 800 *g* for 30 min, and the supernatant was filtered with an 0.45 μm Millipore filter and centrifuged at 17,000 *g* for 90 min [19]. The pellet was suspended in PBS or appropriate culture medium and used for immunofluorescence or incorporation assays in UE6E7T-3 cells. One milliliter of EV was prepared from 100 ml of conditioned medium of U3DT, and the concentration of the resultant EV solution was 0.1 μg/ml, as determined using a NanoDrop 2000 (Thermo Fisher Scientific) using BSA as the standard solution. UE6E7T-3 cells were cultured with 0.3 μg EVs in 2 ml DMEM. After 1 day, the cultured cells were characterized by immunofluorescence staining.

### Immunocytochemistry

Cells cultured on coverslips were fixed in 4% paraformaldehyde in phosphate-buffered saline (PBS), washed in PBS, and then blocked with 1% BSA in PBS. For detection of Rab11, samples were fixed with 10% TCA, washed with 50 mM NH_4_Cl, and permeabilized with 0.05% Triton X-100 [20]. The cells and EVs were subjected to indirect immunofluorescence staining. The following antibodies and fluorescence reagents were used: anti-GPC5 antibody (MAB2607, R&D Systems, Inc.), Alexa Fluor 594 conjugated anti-GPC5 antibody (R&D Systems, Inc.), Alexa Fluor 647 conjugated anti-GPC5 antibody (R&D Systems, Inc.), anti-FGFR1 Xp rabbit monoclonal antibody (D8E4, Cell Signaling Technology), anti-Rab11A antibody (A-6: sc-166912, Santa Cruz Biotech), anti-Rab11 (D4F5)XP rabbit monoclonal antibody (Cell Signaling Technology), anti-acetyl-alpha-tubulin rabbit monoclonal antibody (D20G3, #5335, Cell Signaling Technology), Alexa Fluor 488-conjucated wheat germ agglutinin (WGA; W1126: Invitrogen), anti-CD63 monoclonal antibody (MX-49.129.5: sc-5275, Santa Cruz Biotechnology), anti-CD63 monoclonal antibody (cl 3–13: Fuji Film Co.), anti-ARF6 monoclonal antibody (3A-1: sc-7971, Santa Cruz Biotechnology), anti-ARF6 polyclonal rabbit antibody (20225-1-AP, Proteintech), Alexa Fluor 488-conjugated goat-anti-mouse IgG(H+L), F(ab’)2 fragment (#4408, Cell Signaling), Alexa Fluor 488-conjugated goat-anti-rabbit IgG(H+L), F(ab’)2 fragment (#4412, Cell Signaling Technology), Alexa Fluor 568-labeled donkey-anti-mouse IgG (H+L) (A10037, Invitrogen), Alexa Fluor 594-labeled goat anti-mouse IgG IgG(H+L), F(ab’)2 fragment (A-11020, Molecular Probes, Inc.), and Alexa Fluor 647-conjugated goat-anti-rabbit IgG(H+L), F(ab’)2 fragment (#4414, Cell Signaling Technology). All antibodies were used at a dilution of 1:100 from 0.2–1 μg/ml stock. Immunostained cells or EVs were mounted using ProLong Diamond Antifade Mountant with DAPI (Invitrogen) or Fluoromount/Plus with DAPI (Diagnostic BioSystems) and visualized on a confocal fluorescence microscope (SP8; Leica Microsystems).

### Confocal microscopy

An inverted laser scanning microscope (TCS SP8; Leica Microsystems), equipped with a 63x oil objective (HCPLAPOCS2, NA = 1.4) or a 20x objective (HCPLAPOCS2, NA = 0.75) was used to visualize cells or EVs. For quantitative analysis of fluorescence intensity, fluorescence images were obtained with an extremely light-sensitive HyD detector: fluorescence intensities at 488, 568, 594, and 647 nm were collected in standard mode or photo-counting mode for quantitative detection. Images were 1024 × 1024 pixels and were collected as Z-stacks (Z-step size, 0.24–1.0 μm; zoom, 1.5–10). The sum of fluorescence intensity was calculated from the optimum intensity of z-stack images of each cell, which was expressed as the pixel sum of each cell, using the LASX software (v.3.4, 18368.2) [21]. For quantitative detection, samples were stained under the same conditions, and immunofluorescence images were obtained under the same light and detector conditions on the same day. The background pixel sum of each cell was below 5%.

### Flow cytometry

UE6E7T-3 or U3DT cells treated with or without siRNA-RAB11A for 3 days were harvested with trypsin and were fixed with 4% paraformaldehyde-PBS. The cells permeabilized with 0.05% Triton-X100 were suspended in 0.1% BSA-PBS at a concentration of 5–10 × 10^5^ cells/ml. One hundred microliters of each cell suspension were mixed with 5 μl antibody diluted 20-fold in PBS containing 5% FBS (5% FBS-PBS). After incubation overnight at 4°C, the cell suspension was washed twice with 5% FBS-PBS and shaken with secondary antibodies for 4 h at 4°C.

Data acquisition from 10,000 cells per sample was performed on a FACSAria (BD Biosciences), and the resultant data were analyzed using the FlowJo software (TOMY Digital Biology) [22]. The antibodies used in this test were as follows: anti-GPC5 mouse antibody, anti-Rab11A rabbit antibody, Alexa Fluor 488-conjugated anti-mouse antibody, and Alexa Fluor 594-conjugated anti-rabbit antibody.

### siRNA treatment

U3DT cells were seeded in 6-well chamber slides (5 × 10^3^ cells per well) and cultured in DMEM containing 10% FBS. The following day, cells were transfected with 100 or 200 nM Accell Human Rab11A siRNA SMARTPool, 40 nM Accell Human GPC5 siRNA SMARTPool, or a non-targeting control siRNA (GE Healthcare Dharmacon Inc. USA) with 0.1% FBS, as described previously [3]. After 72 h, cells in each well were fixed with 4% paraformaldehyde and characterized by immunofluorescence or by flow cytometry.

### Transmission electron microscopy

A 5 μl aliquot of an EV sample was applied to a glow-discharged carbon film grid, stained with 1% (w/v) uranyl acetate solution, and rinsed with distilled water. Grids were examined using a Hitachi H-7650 electron microscope with an acceleration voltage of 80 kV. EV diameters were calculated from ten representative images using the Hitachi EM viewer Ver 03.01 software.

### Wound healing assay

A total of 10,000 cells were seeded into each well of a 4-well chamber slide (Invitrogen) or each well of a culture insert in a 35mm μ-Dish (80206, ibidi, LLC, Verona, WI). For knockdown of GPC5, cells were cultured for 48 h and then transfected with 40nM siRNA-GPC5, or non-targeting siRNA (Accell human SMARTPool), or cultured in Dharmacon Medium as a mock transfection (mock) for 72 h. After cells reached confluence, 5μg/ml mitomysin-C (Invitrogen) was added to inhibit cell proliferation and removed by washing after 2 h. Subsequently, the insert of a *μ*-Dish was removed, and cells were incubated in an appropriate medium with or without 25 nM basic FGF (Invitrogen, USA) at 37 °C for ca. 23 h in an incubator containing 5% CO_2_.

### Statistical analysis

Values were expressed as means ± S.D. of more than three experiments. Differences between means of individual groups were assessed by Student’s two-tailed unpaired t-test. *p* < 0.05 was considered statistically significant. n.s., not significant, ***, *p* < 0.001; **, *p* < 0.01; and *, *p* < 0.05.

## Results

### GPC5 is dramatically overexpressed in association with transformation of UE6E7T-3 cells

In a previous study, we performed whole-transcriptome analysis of the human mesenchymal stem cell line, UE6E7T-3, over the course of transformation. The results revealed that GPC5 was dramatically overexpressed in association with transformation [3]. Fig 1 shows glypican expression at four stages of culture spanning 295 population doubling levels (PDLs) in the previous study. GPC5 was expressed at low levels at the early stage (PDL 60–90, sample U3-A) and overexpressed at the late stage (PDL 231–295, sample U3DT) of long-term culture with increasing the proliferation as shown in the previous report [3]. Other GPCs (GPC1 to GPC4 and GPC6) were expressed at low levels throughout the culture period.

### Cell-surface GPC5 colocalizes with FGFR1, Rab11, and ARF6

**Fig 2:**
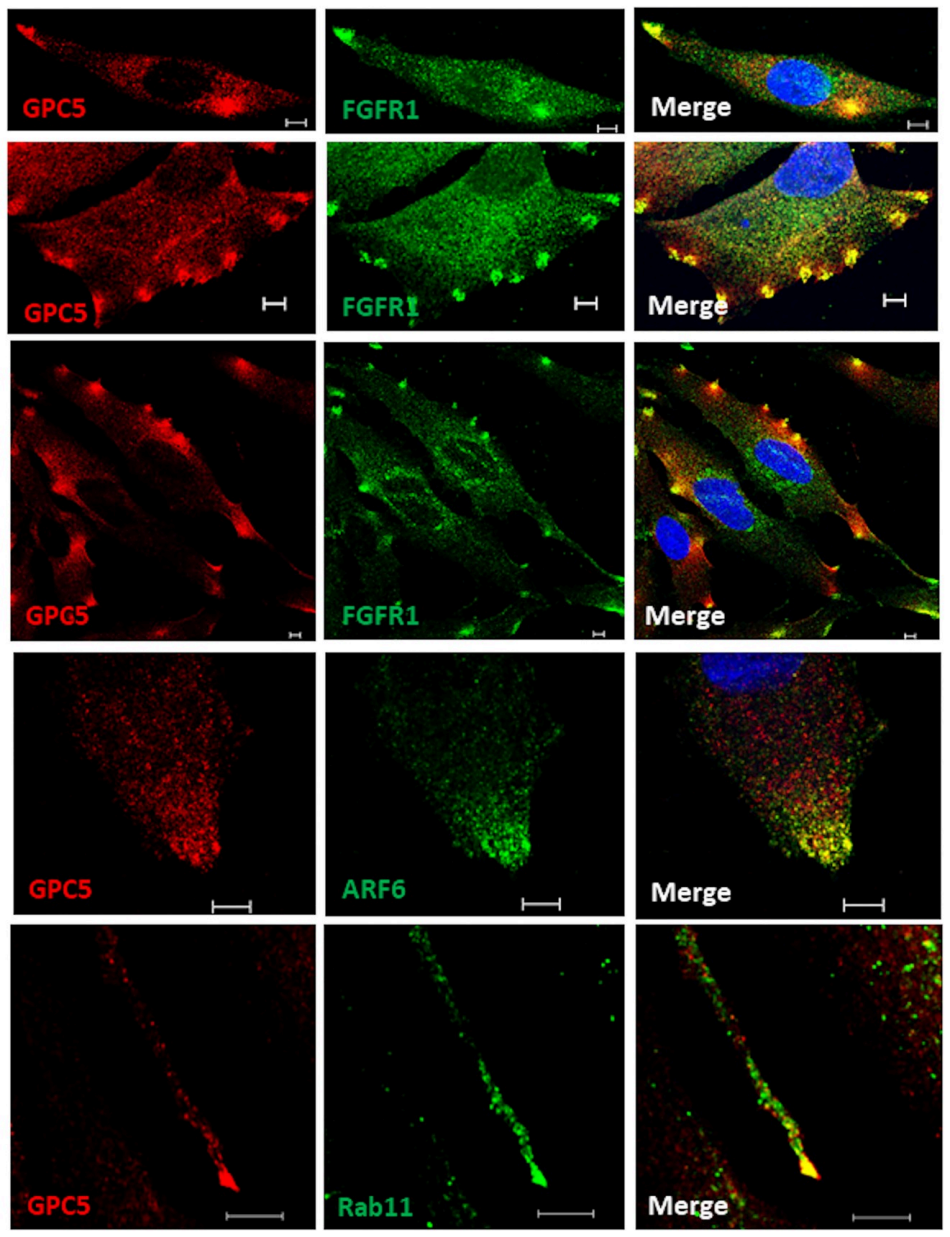
GPC5s localize to the leading edges of migrating cells along with FGF receptor1, Rab11, or ARF6. Double immunofluorescence labeling of U3DT cells with anti-GPC5 (red) and anti-FGFR (green) (top three lines), with anti-GPC5 (red) and anti-ARF (green) (fourth line), or with anti-GPC5 (red) and anti-Ra11A (green) (bottom line) antibodies. Positive spots (yellow) in each merged image were clearly visible in U3DT cells. Scale bar, 5 μm.

The FGF–FGFR signaling pathway is involved in cell migration during wound healing [23], and chondroitin and dermatan sulfate or SDC4 modulate FGF-induced cell migration [24, 25]. To investigate the interaction between GPC5 and FGFR1, we used immunofluorescence microscopy to examine the localization of endogenously expressed GPC5 and FGFR1 in U3DT cells under steady-state culture conditions. In interphase cells, strong staining patterns of colocalization of GPC5 and FGFR1 were observed in the perinuclear region, and a punctate vesicular pattern was dispersed throughout the cell surface. Puncta were detected at the leading edges, such as the tip at the front of an elongating cell (Fig 2, top panel), blunt-ended protrusions at the front of lamellipodia (Fig 2, second panel), and the region adhered to the substratum of the glass coverslip when both daughter cells separated during cytokinesis, as if they might aid in the mechanical separation of the two daughter cells (Fig 2, third panel). These patterns suggest that GPC5 modulates FGF-mediated cell migration. To confirm the specificity of immunofluorescence analysis, UE6E7T-3 and U3DT cells were stained with and without an anti-GPC5 antibody (S1 Fig). U3DT cells were stained strongly by the anti-GPC5 antibody, but UE6E7T-3 cells were not (S1B and S1C Fig), which is consistent with the data presented in Fig 1. In addition, Rab11 and ARF6 GTPases have been implicated in regulation of cell motility [26]. In particular, they regulate the recycling of plasma membrane receptors. Hence, we tested whether GPC5 also interacts with ARF6 or Rab11 in U3DT cell migration. As shown in Fig 2 (two bottom panels), both ARF6 and Rab11 were also colocalized with GPC5 at the leading edge of migrating U3DT cells. Based on the results, it is possible that GPC5 and FGFR1 are transported to the plasma membrane by the endocytic recycling pathway and may promote FGF-mediated cell migration.

### Subcellular localizations of GPC5 during cell division

**Fig 3:**
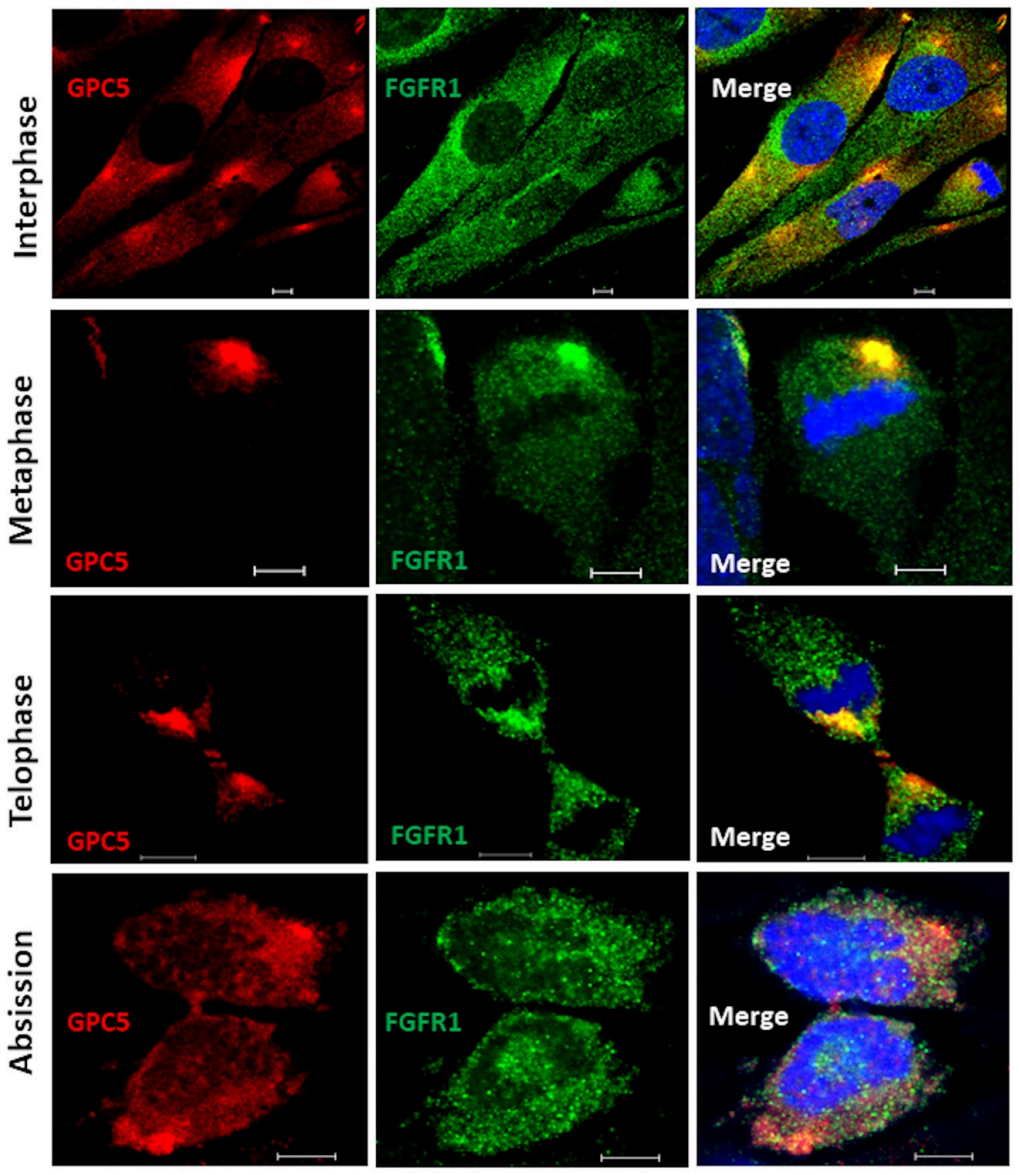
Dynamic localization of GPC5 during mitosis and cytokinesis. U3DT cells at different stages of mitosis and cytokinesis were fixed but not permeabilized, stained with anti-GPC5 (red) and anti-FGFR1 (green) antibodies, and counterstained with DAPI stain (blue). Yellow represents the degree of colocalization. Scale bar, 5 μm.

**Fig 4:**
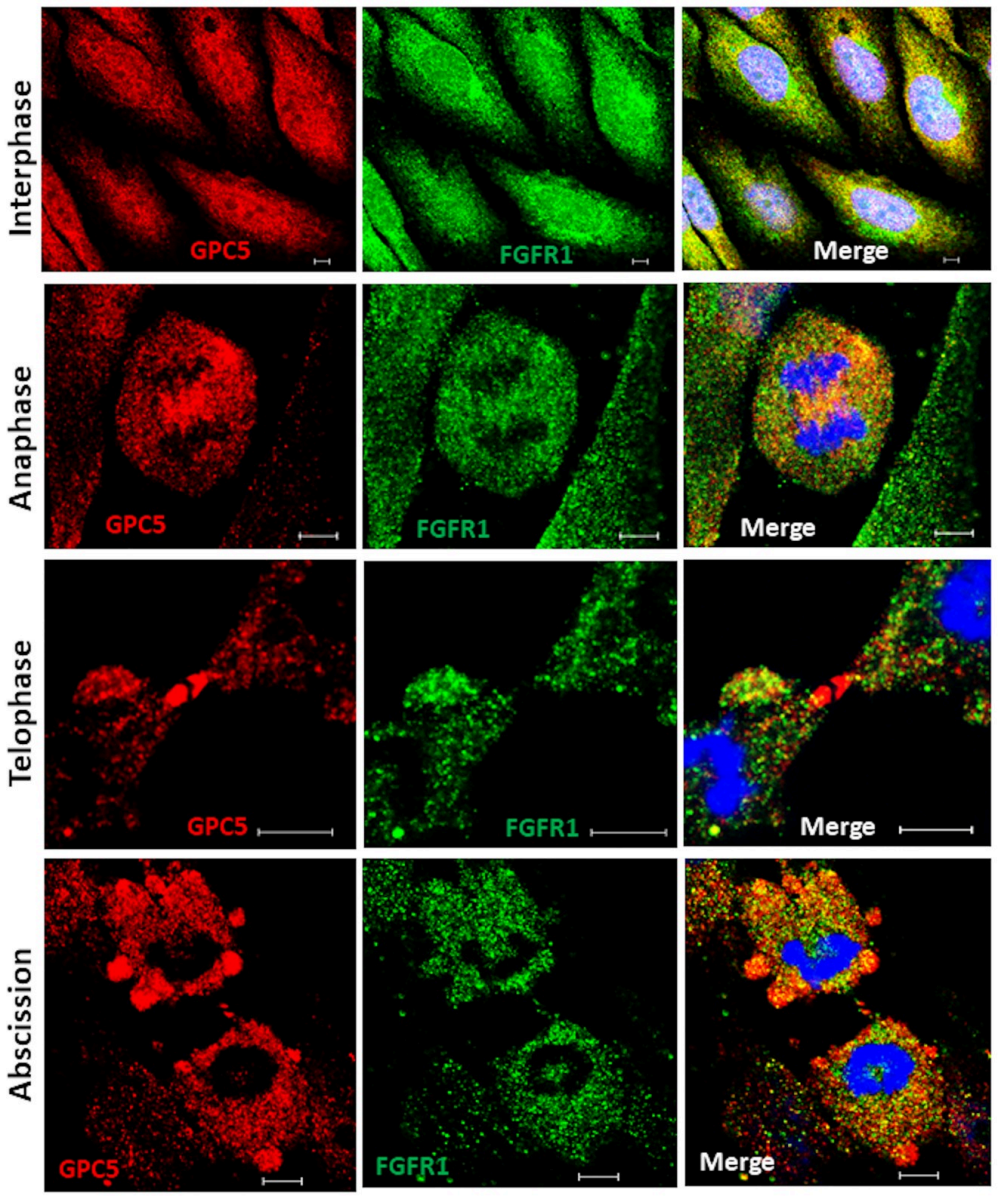
Dynamic localization of GPC5 during mitosis and cytokinesis with permeabilized cells. Cells expressing the same marker as in Fig 3. Scale bar, 5 μm.

Although GPC5 was thought to localize to the plasma membrane during interphase, its possible interactions with ARF6 and Rab11 suggest that the subcellular localization of GPC5 differs according to the cell cycle phase. To test the interaction between GPC5 and FGFR1 during cell division, we first examined the distributions of endogenous membrane GPC5 and FGFR1 in non-permeable U3DT cells by immunostaining. Double staining for GPC5 and FGFR1 revealed that in interphase cells, the two proteins were colocalized at perinuclear regions and leading edges (Fig 3). By contrast, the staining patterns in mitotic cells were quite distinct from those in interphase cells. In rounded metaphase cells, strong staining was observed in one or two regions (Fig 3, second line); during mitosis, staining was detected adjacent to the cleavage furrow (Fig 3, third line). In later stages of cytokinesis, strong staining for both GPC5 and FGFR1 accumulated at the intercellular bridge. At the last step of cytokinesis (abscission), strong GPC5 was also detected in bleb protrusions of both daughter cells (Fig 3, abscission). On the other hand, in the cells permeabilized after fixation (Fig 4), both GPC5 and FGFR1 exhibited only punctuated staining patterns dispersed throughout interphase cells. At anaphase, however, GPC5 accumulated at the equatorial plane of the cell cortex with FGFR1, and at telophase, GPC5 staining became distinctly concentrated around the central spindle and intercellular bridge (Fig 4, third line), whereas FGFR1 staining dispersed into puncta throughout the cell. Similarly, in the last stages of cytokinesis, GPC5 accumulated at high levels in membrane surface blebs, whereas FGFR1 did not. These results indicate that GPC5 may be transported and play significant roles, some of which involve an interaction with FGFR1, at both the midbody and abscission.

### Midbody localization of GPC5

**Fig 5:**
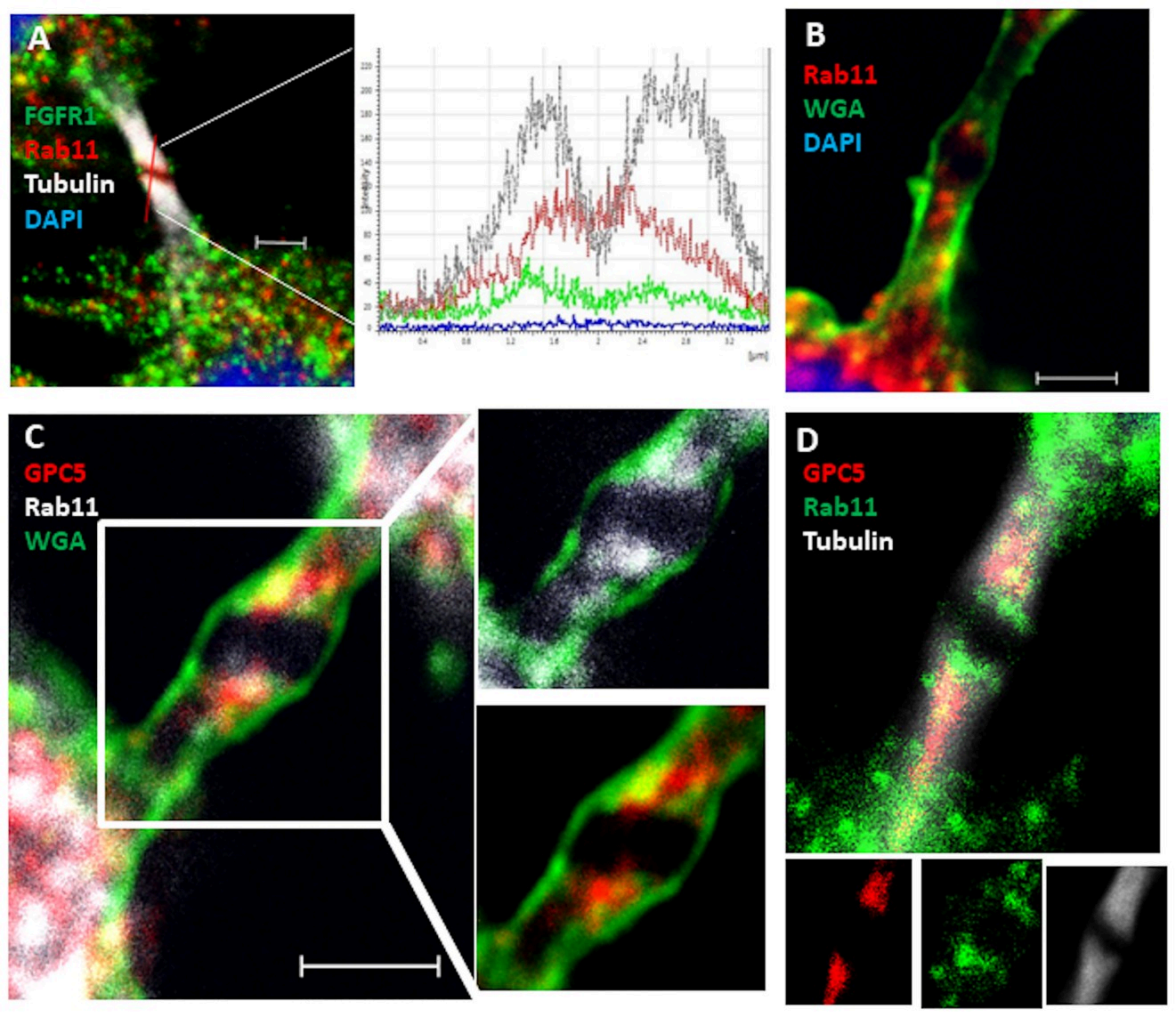
GPC5 localizes to the intercellular bridge. (A) Rab11 localized on both sides (red) of the midbody dark zone and partially overlapped with microtubules (gray). Localizations of proteins on midbody microtubules were determined and compared by line scans. Microtubules and Rab11 peaked at the same positions, where the microtubule signal was high and the FGFR signal (green) was low. (B) Rab11 localization (red) adjacent to the midbody. Plasma membrane was stained with WGA (green). (C) GPC5 (red) colocalized with Rab11 (gray) at the midbody. The plasma membrane was stained with WGA (green). (D) GPC5 (red) colocalized with Rab11 (green) on midbody microtubules (gray). Scale bar, 2 μm.

The considerable accumulation of GPC5 at the midbody during telophase was very interesting to us. Large numbers of components are localized within the midzone during telophase [27]. Among them, Rab11, one of the best-studied Rab small G proteins, associates with recycling endosomes and traffics into the midbody during cytokinesis [28, 29]. To explore the significance of GPC5 localization at the midbody, we examined the colocalization of GPC5 with tubulin or Rab11. Two faint bands outside the dark zone, where tubulin staining did not appear, represented Rab11. By contrast, Rab11 was highly enriched within punctate structures in the proximity of the cleavage furrow, where FGFR1 was also present, although it appeared not to associate with Rab11 (Fig 5A). To further confirm this observation, we used fluorescent WGA [30], a membrane marker that distinguishes the cell-surface membrane from cytosolic components. Rab11 staining could also be observed throughout the intercellular bridge but not at the center of the bulge (dark zone) (Fig 5B). Interestingly, GPC5 also localized there in a pattern that partially overlapped with that of Rab11 (Fig 5C). The spatial distributions of GPC5, Rab11, and microtubules are shown in Fig 5D, which confirms the partial overlap of GPC5 with Rab11. These results demonstrate that both GPC5 and Rab11 localize to the intercellular bridge during telophase, but their interaction remains obscure.

### Bleb dynamics in cytokinesis

**Fig 6:**
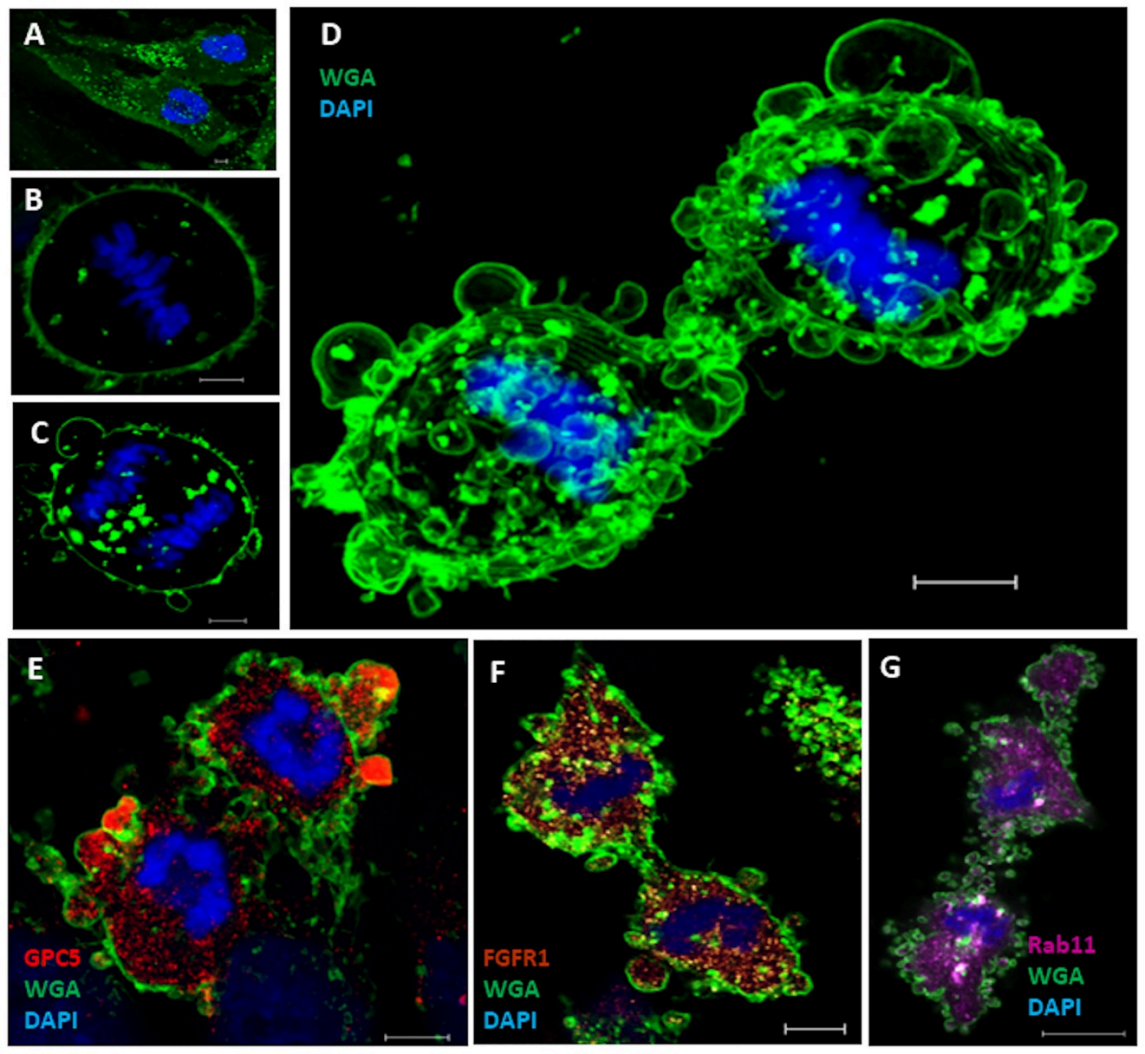
Localization of GPC5 in blebs of U3DT cells at telophase. (A‒D) Images of staining with Alexa Fluor 488-conjugated WGA (green) and DAPI (blue) at interphase (A), metaphase (B), and anaphase and telophase (C, D). (D) Maximum projection of 15 Z-staged-images stained with Alexa Fluor 488-WGA and DAPI. (E) U3DT cell at telophase, stained with anti-GPC5 antibody (red), Alexa Fluor 488-WGA (green), and DAPI (blue). (F) Blebs of U3DT cell at telophase stained with anti-FGFR1 (brown), Alexa Fluor 488-WGA (green), and DAPI (blue). (G) Blebs of U3DT cells at telophase stained with anti-Rab11 rabbit antibodies (magenta), Alexa Fluor 488-WGA (green), and DAPI (blue). Scale bar, 5 μm.

Membrane blebs have been observed at the poles and midbody of dividing cells [31]. In the present study, U3DT cells spontaneously exhibited dynamic membrane blebs during later stages of mitosis (Figs 3 and 4). Fig 6A–6D show WGA fluorescence staining of U3DT cells during mitosis; membrane blebbing occurred over the entire surface at telophase, similar to exocytic bursts of membrane (Fig 6D). In some blebs, GPC5 was strongly fluorescently labeled (Fig 6E), whereas FGFR1 and Rab11 staining was of similar intensity in blebs and in the cytosol inside the surface membrane (Fig 6F and 6G).

**Fig 7:**
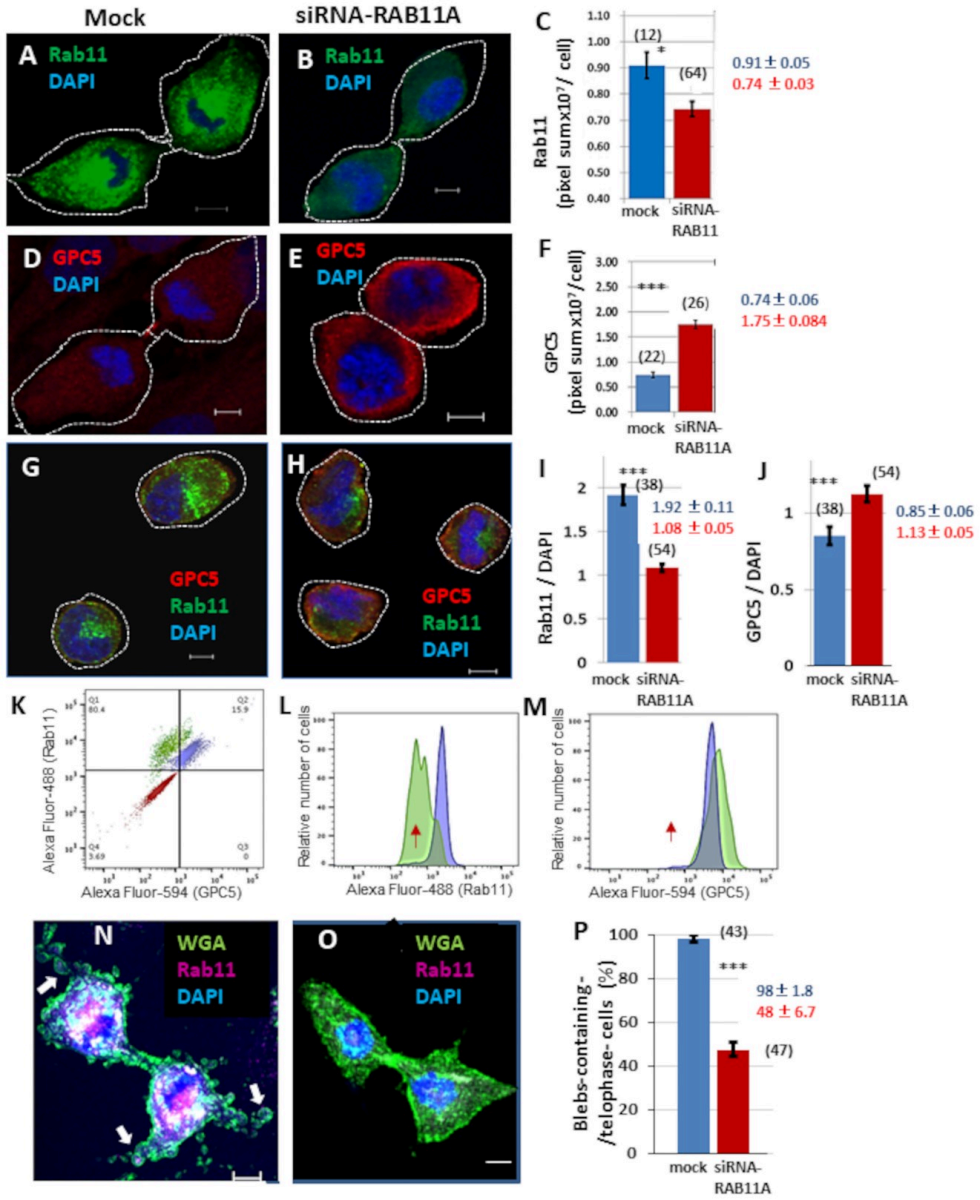
Localization of GPC5 and Rab11 in U3DT cells treated with RAB11A-siRNA. Immunofluorescence images of cells were obtained using a Leica SP-8 confocal microscope, and the pixel sum was estimated with the SP8 software (RAS X). (A,B) Images of Rab11 in untreated (A) and RAB11A-siRNA-treated U3DT cells (B). (C) Quantitative analysis of U3DT cells not treated (blue) or treated (red) with 100 nM RAB11A-siRNA. (D,E) Images of GPC5 in U3DT cells not treated (D) or treated (E) with 100 nM RAB11A-siRNA. (F) Quantitative expression of GPC5 in U3DT cells not treated (blue) or treated (red) with 100 nM RAB11A-siRNA. (G,H) Images of GPC5 (red) and Rab11 (green) in trypsinized-U3DT cells not treated (G) or treated (H) with RAB11A-siRNA. (G‒J) Immunofluorescence images (G,H) and the pixel sum (I,J) of trypsinized cells were obtained using a Leica SP-8 immunofluorescence microscope the same as in C and F. (I) The mean intensity (pixel sum) of Rab11 relative to that of DAPI is shown for each untreated (blue) and RAB11A-siRNA-treated U3DT cell (red). (J) The mean fluorescence intensity (pixel sum) of GPC5 relative to that of DAPI staining per cell is shown for each untreated (blue) or RAB11A-siRNA-treated U3DT cell (red). (K‒M) FACS analysis. (K) Contour display of merged three images. U3DT cells were stained with (blue) or without (red) anti-GPC5 and anti-Rab11 antibodies. RAB11A-siRNA-treated cells were stained with anti-GPC5 and anti-Rab11 antibodies (green). (L) Histogram of Alexa Fluor 488 fluorescence intensity (Rab11) of RAB11A-siRNA-treated cells (green) or not treated cells (blue). (M) Histogram of Alexa Fluor 594 fluorescence intensity (GPC5) of RAB11A-siRNA-treated cells (green) or not treated cells (blue). The same preparation was used for immunofluorescence (G, H) and for FACS analyzes (L, M). Red arrows indicate the peak position of immunostaining control cells (L, M). (N, O) Immunofluorescence images of Rab11 (red) and WGA (green) in not treated cells (N) and RAB11A-siRNA-treated cells (O). (P) The mean distribution of telophase cells with three or more blebs (>ca. 2 μm diameter) in telophase are shown for not treated cells (blue) and RAB11A-siRNA-treated cells (red). The numbers in parentheses in Figure (C, F, I, J and P) are the number of images. White arrows indicate blebs in a telophase cell (N). Scale bar, 5 μm.

The role of blebbing in cytokinesis remains obscure, but it has been speculated that blebbing might contribute to separation of daughter cells via the vigorous motile activity induced by actomyosin contractile system and membrane reservoirs [32]. Blebbing appears to correlate with membrane transport. Therefore, we investigated whether the recycling endosome marker Rab11 is present in blebs during cytokinesis. Rab11 was uniformly distributed in blebs as well as cytoplasm (Fig 6G). Double labeling of GPC5 and Rab11 revealed strong punctate GPC5 labeling at the leading edges of blebs, whereas Rab11 was found partial localization in blebs, suggesting that the two proteins partially overlap in blebs (S2E and S2F Fig). To further confirm this, we examined the effects of Rab11 depletion on GPC5 localization and bleb formation during cytokinesis. Immunostaining revealed a significant reduction in Rab11 intensity (Fig 7A–7C, 20% decrease), whereas a considerable increase in GPC5 intensity was detected following treatment with RAB11A-siRNA (Fig 7D–7F, 200% increase). To eliminate non-specific adsorption of antibodies, trypsinized cells were tested. Double labeling of cells in suspension (trypsin treatment) with GPC5 and Rab11 yielded results similar to those obtained in adhered cells (Fig 7G–7J and S3 Fig). Rab11A-knockdown U3DT cells showed a decrease in the Rab11 intensity (Fig 7G) and an increase in the GPC5 intensity (Fig 7J) which were also confirmed by FACS analysis (Fig 7K–7M). In addition, RAB11A-siRNA-treated cells exhibited cytokinesis defects, such as formation of too few membrane blebs (Fig 7N–7P).

### GPC5 is released into the conditioned medium

**Fig 8:**
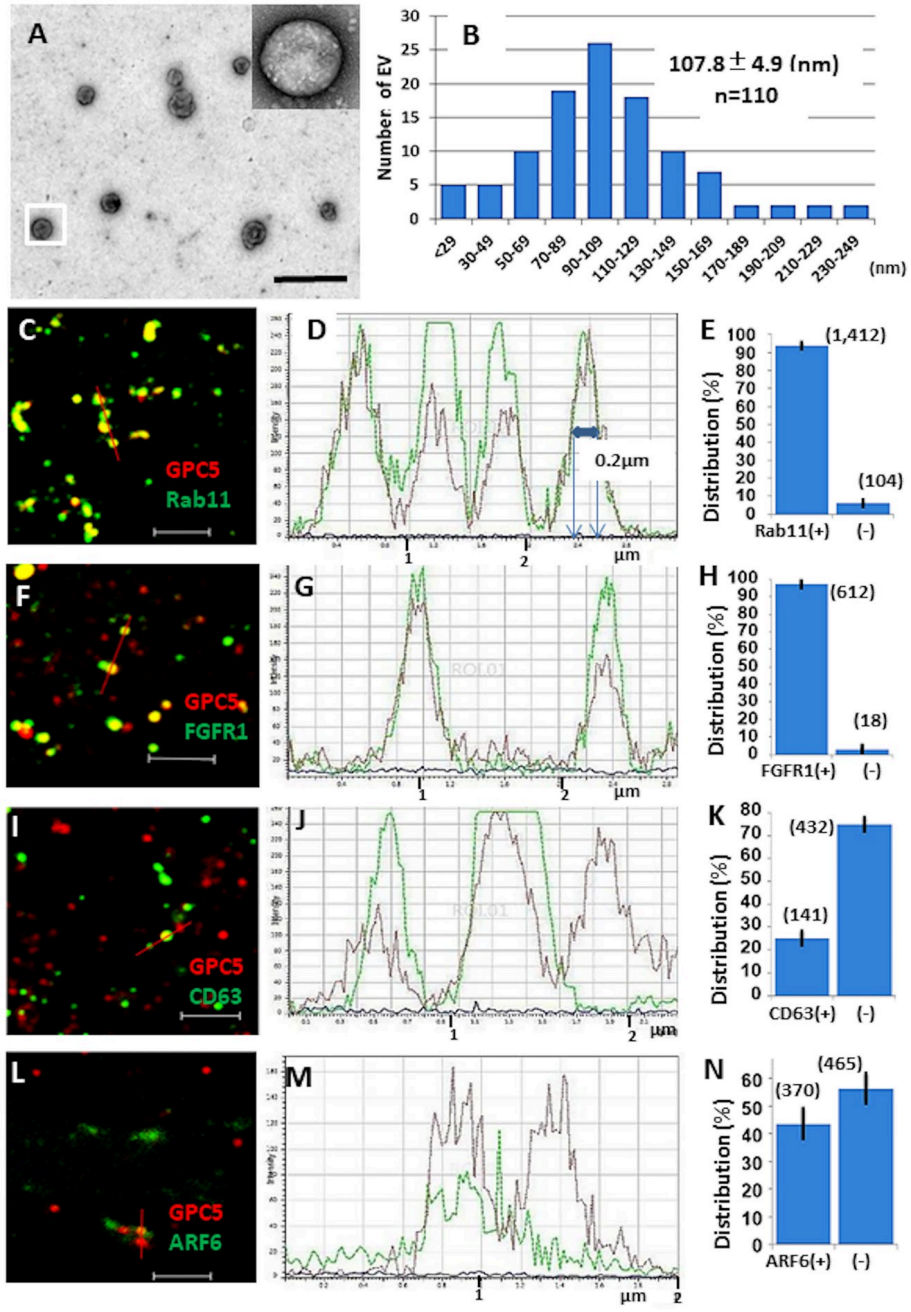
GPC5 is present on EVs from U3DT cells. (A) Electron microscopic image of negative-stained EVs. The inset shows a magnification of the boxed EV. (B) Size distribution of vesicles in EV preparations measured by image software (n = 110). (C) (F) (I) (L) GPC5-immunostained EVs (red) stained for Rab11 (green) (C), FGFR1 (green) (F), CD63 (green) (I), and ARF6 (green) (L). In (I) and (L), an Alexa Fluor 647- conjugated anti-GPC5 antibody was used to detect of GPC5-ositive particles. (D) (G) (J) (M) Line scan determination of the red bars in (C), (F), (I), and (L): GPC5 (red), others (green). (E) (H) (K) (N) Distribution of GPC5-positive particles identified by scan determinations in (D), (G), (J), and (M). n = 1,516, (E), 630 (H), 573 (K), and 835 (N). Scale bars: (A), 500 nm; (C), (F), (I), (L), 2 μm.

Circulating exosomes positive for GPC1 have been isolated from the serum of patients with pancreatic ductal adenocarcinoma [33]. Therefore, we assessed whether GPC5 could be detected in the conditioned medium of U3DT cells. We isolated EVs from the conditioned medium of U3DT cells by differential ultracentrifugation and filtration. The EV population was mixed, and electron microscopy revealed a size distribution with a mean diameter of 108 nm (Fig 8A and 8B). Otherwise, analyses of immunofluorescence images indicated that EVs had a diameter of 0.2 μm, which is beyond the resolution of SP-8 microscope under a condition of these observations (Fig 8D). EVs immunostained with anti-Rab11 (Fig 8C), anti-FGFR1 (Fig 8F), anti-CD63 (Fig 8I), and ant-ARF6 (Fig 8L) antibodies exhibited significant colocalizations with GPC5, as supported by line scan determination (Fig 8D, 8G, 8J, and 8M). Additionally, 93% and 97% of GPC5-containing EVs colocalized with Rab11 and FGFR1, respectively (Fig 8E and 8H), whereas 25% and 44% of GPC5-containing EVs colocalized with CD63 and ARF6, respectively (Fig 8K and 8N). These results indicate that GPC5-containing EVs generally contain Rab11 or FGFR, but do not often contain CD63 or ARF6.

**Fig 9:**
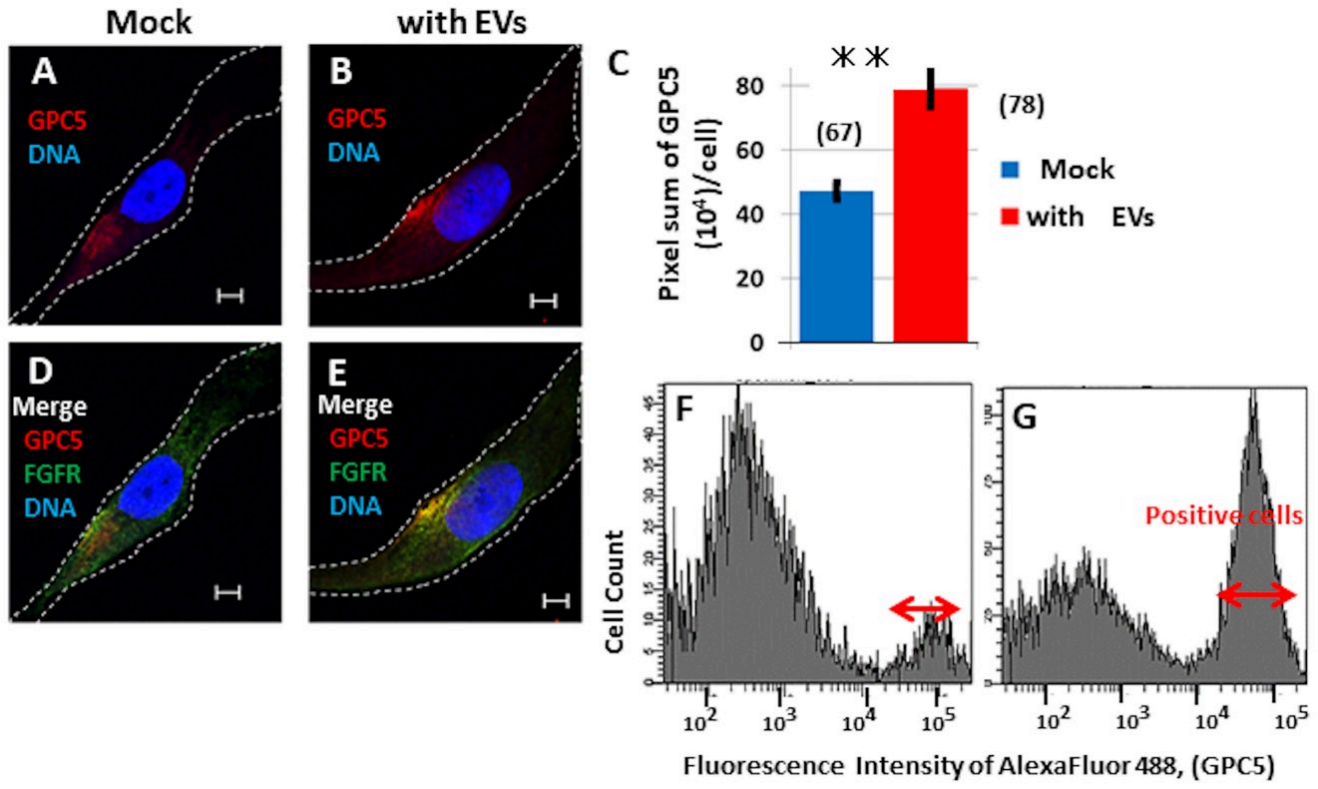
EVs are incorporated to UE6E7T-3 cells. (A, D) Images of control cells. (B, E) Images of EV-treated cells. (D, E) Merged images of UE6E7T-3 cells (D) and EV-treated cells (E) were stained for GPC5 (red) and FGFR1 (green). (C) Quantitation of GPC5 (pixel sum per cell) in control UE6E7T-3 cells (blue) or cells cultured with EVs for 1 day (red). n = 67 (blue) and 78 (red). (F, G) FACS pattern of GPC5 in UE6E7T-3 cells (F) and in cells cultured with EVs for 1 day (G). ⇔ indicates GPC5-positive cells. Scale bar, 5 μm.

Previously, diverse biological functions have been attributed to EVs [34]. To examine the functional interactions of EVs with cells, we tested U3DT-EVs for their ability to deliver GPC5 to UE6E7T-3 cells, the parental cell line of U3DT, which expresses very little if any GPC5 (Fig 9A and 9D and S1B Fig). After culture of UE6E7T-3 cells with U3DT-EVs for 25 h, cells double-stained with anti-GPC5 and anti-FGFR1 antibodies were positive for GPC5 in the perinuclear region (Fig 9B), and the GPC5 signal overlapped with the FGFR1 signal in this region (Fig 9E). Quantitative analysis of GPC5 in a UE6E7T-3 cell was performed using a Leica SP-8 immunofluorescence microscope as described in the Method (Fig 9D and 9E). The pixel sum of GPC5 indicated that particles taken up by UE6E7T-3 cells (Fig 9C). Flow-cytometric analysis also revealed extensive uptake of the U3DT-EVs by UE6E7T-3 cells (Fig 9F and 9G). The results demonstrate that EVs released by U3DT cells were taken up by UE6E7T-3 cells.

### GPC5 promotes migration of U3DT cells

**Fig 10:**
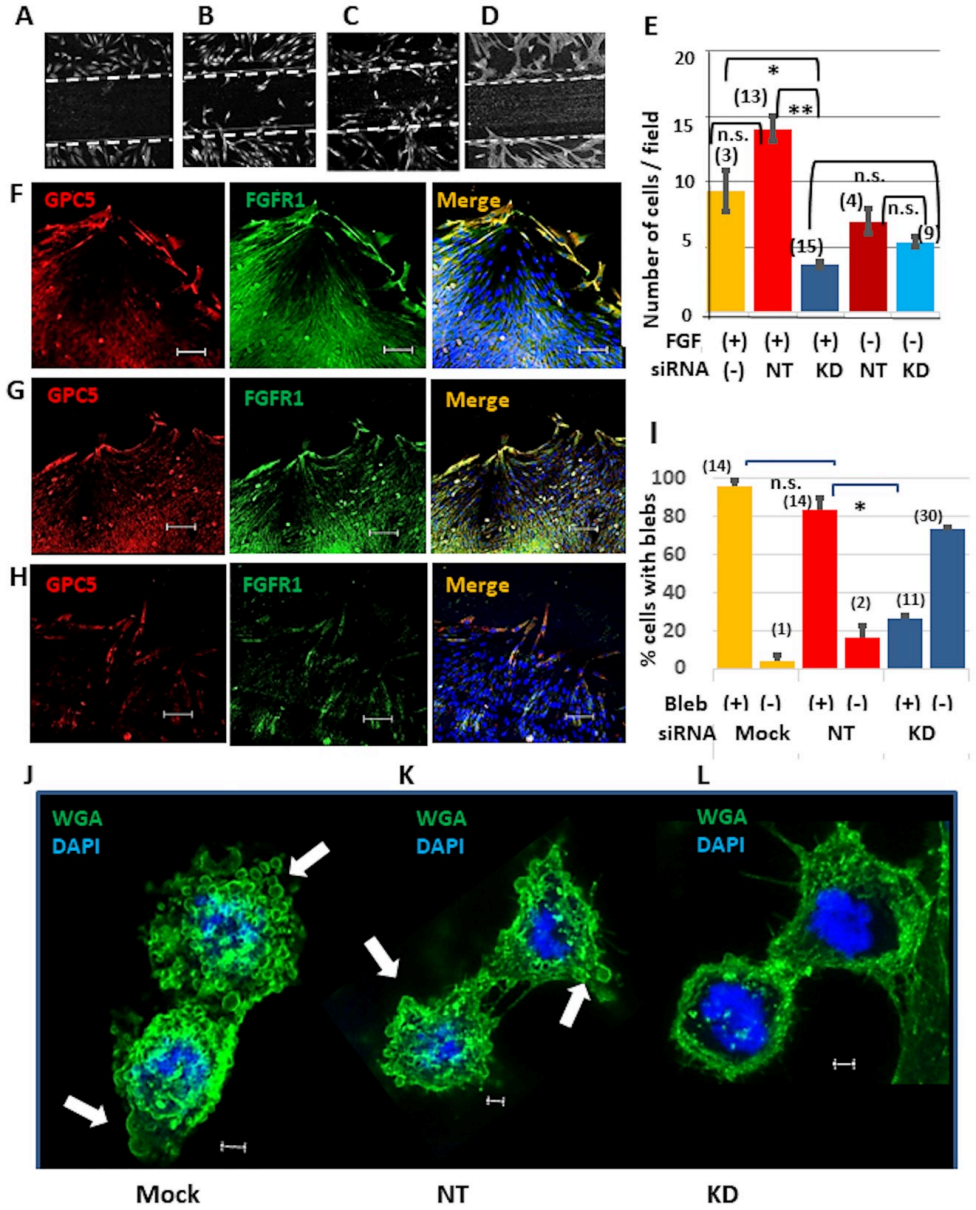
Wound healing of U3DT cells. (A) Control cells fixed immediately after scratching. The wide of the scratched area was ca. 500μm. (B‒D) Mock (B), non-targeting siRNA (NT)-treated (C), and GPC5-siRNA (KD)-treated (D) cells were cultured in appropriate medium containing 25 nM FGF2 for 23 h after scratching. Cells were fixed and immunofluorescence images were acquired using a Leica SP-8 microscope equipped with a 20x objective. (E) The number of cells that moved into the scratched area or the removed insert area was counted after incubation of control (without siRNA) cells (yellow), NT cells (red), and KD cells (blue) in appropriate medium containing (red and blue columns) or lacking (brown and sky blue columns) FGF2 at 37°C for 23 h. (F‒H) Immunofluorescence images of cells treated with mock (F), non-targeting siRNA (G), or GPC5-targeting siRNA (H) for 72 h after removing an insert from a *μ*-Dish. Cells were stained with anti-GPC5 (red) and anti-FGFR1 (green) antibodies. (I) Quantification of the percentage of cells with blebs at telophase. Control (mock) cells (yellow), NT cells (red) and KD cells (blue) were incubated in appropriate medium containing FGF2 at 37°C for 23 h. (J‒L) Immunofluorescence images of mock (J), non-targeting siRNA-treated (K), and GPC5-siRNA-treated (L) cells after 72 h. Cells were stained with WGA (green). White arrows indicate blebs in a telophase cell (J, K). The numbers in parentheses in (E, I) are the number of images observed. Scale bar: (F‒H), 100 μm; (J‒L), 2 μm.

GPC5 localizes to lamellipodia and blebs, therefore, its contribution to cell migration was examined using the scratch assay. U3DT cells were transformed with GPC5-targeting or non-targeting control siRNA. The DNA synthesis inhibitor mitomysin-C was used to prevent cell proliferation confounding the migration assay. The number of cells that migrated from the edge of the scratch in the presence or absence of FGF2 was counted (Fig 10A–10E). Migration of GPC5-knockdown cells (Fig 10D and 10E, blue and sky blue columns) did not significantly differ in the presence or absence of FGF2, and there was no significant difference in migration between GPC5-knockdown cells and non-targeting siRNA-treated cells cultured without FGF2 (Fig 10E, sky blue and brown columns). However, FGF2 considerably stimulated migration of non-targeting siRNA-treated cells (Fig 10C and 10E, red column), but not of GPC5-knockdown cells (Fig 10D and 10E, blue column). This indicates that FGF2 stimulation of U3DT cell migration is completely dependent on GPC5, because GPC5 knockdown eliminated the response to FGF2. This result is similar to that observed upon heparinase III treatment of heparan sulfate proteoglycans (HSPGs) [35]. To determine whether GPC5 is upregulated in U3DT cells to drive cell migration, we examined its distribution in migrating cells. The level of GPC5 was markedly increased in cells migrating from the scratch edge and those located at the scratch edge (Fig 10F and 10G). FGFR1 also colocalized with GPC5, while GPC5-knockdown cells did not show any increase in GPC5 expression (Fig 10H). Taken together, these results suggest that GPC5 upregulation is required for FGF2-dependent migration of U3DT cells during wound healing. On the other hand, GPC5-knockdown cells in steady-state culture displayed less GPC5 staining than untreated and non-targeting control (NT) siRNA-treated cells, but staining of FGFR1, ARF6, and Rab11 was unaffected (S4 Fig). Consequently, colocalization of GPC5 with FGFR1, ARF6, and Rab11 was not observed at the tips of elongated cells which were treated with GPC5-siRNA (S4 Fig).

GPC5 knockdown also likely affects bleb formation (Fig 10I–10L). Although mitosis was observed in GPC5 siRNA-treated cells similar to untreated cells, few blebs appear in the former cells and those that did were much smaller (Fig 10L). Untreated and non-targeting siRNA-treated cells showed high percentages of cells with blebs (>80%) (Fig 10I, orange and red columns), whereas the number of GPC5 siRNA-treated cells with blebs were quite low (<30%) (Fig 10I, blue column). This suggests that U3DT cell migration involved blebbing as showed on mouse mesenchymal precursor cells [36]. These results suggest that GPC5 plays an important role in dynamic cell migration.

## Discussion

The results of this study reveal novel subcellular localizations of GPC5 in association with dynamic cell motility. Although understanding the detailed functions of GPC5 will require further studies at the molecular level, we clearly demonstrated that GPC5 contributes to cell motility. Our recent gene expression analysis of U3DT cells revealed that GPC5 colocalizes with Ptc1 in the perinuclear region. In this study of the subcellular distribution of GPC5, we found that the protein markedly accumulated at the leading edge of cell migration, intercellular bridge, and bleb protrusions during cytokinesis, as well as in EVs released into the conditioned medium.

Cell-surface HSPGs contribute to signal transduction as co-receptors, as well as to the stable retention of multiple growth factors. To date, the contribution of HSPGs to cell migration has mostly been studied in regard to the involvement of SDC4 in FGF-induced migration. SDC4 mediates the matrix-induced protein kinase Cα(PKCα)-dependent activation of Rac1 (Rho family of GTPase) and localizes Rac1 to the leading edge of migrating cells, thereby promoting migration [37]. However, SDC4-induced cell migration can occur even in the presence of dominant-negative FGFR1 [35]. In this study, we demonstrated strong colocalization of GPC5 and FGFR1 at the leading edges of migrating cells in response to unknown stimuli. This phenomenon may be induced by the FGF-stimulated FGFR1 signaling pathway, in which activated FGFR1 stimulates P13-kinase, resulting in Rac1 activation and leading in turn to migration [38]. The pathway is different from the SDC4-induced PKCα-dependent signaling pathway [39]. Thus, we speculate that GPC5 promotes the migration of U3DT cells via the FGFR1 signaling pathway.

Further evidence that GPC5 also colocalizes with Rab11 or ARF6 at the leading edge of migrating cell suggests recycling endosomal dynamics in U3DT cells (Fig 2). Both Rab11 and ARF6 are well known for their roles in regulating vesicular transport and cytoskeletal dynamics [40–42]. In cultured cells, Rab11 transports membrane cargos to and from recycling endosomes on the way to the plasma membrane [20, 43], whereas ARF6 induces the formation of large lamellipodia and promotes cell migration [44], suggesting the involvement of the Rab11 or ARF6 recycling pathways in the transport of GPC5 and FGFR1 in U3DT cell migration.

This study demonstrated an important function of GPC5 in FGF-induced cell migration. GPC5 accumulated at the leading edge of migrating cells. Knockdown of GPC5 using siRNA delayed cell migration and remarkably decreased the GPC5 level in cells. In addition, the GPC5 level was increased in migrating cells. These results suggest that GPC5 upregulation is required for cell migration, and that GPC5 functions in U3DT cell migration. Although GPC5 is not absolutely required for cell migration, it might be needed to stimulate dynamic cell migration.

Interestingly, we found that GPC5 was enriched in the intercellular bridge connecting the daughters during division of U3DT cells. During cytokinesis, many factors necessary for abscission localize to the cleavage furrow and midbody through a variety of pathways, including endosomal recycling. The Rab11-dependent recycling pathways influence a wide range of cell-surface proteins. Because Rab11-positive vesicles separate from Rab11-containing recycling endosomes and traffic to the cleavage furrow and midbody, we first sought to determine whether GPC5 associates with Rab11. Three lines of evidence suggest this possibility. First, GPC5 colocalizes with Rab11 at intercellular bridges in late telophase. GPC5 is probably transported to the intercellular bridge in association with Rab11-endosomes. Second, depletion of Rab11 using RAB11A-siRNA gave rise to accumulation of GPC5 inside the cell, as demonstrated by immunostaining and FACS analysis. This is consistent with previous studies showing that Rab11 depletion inhibits exocytic events of recycling vesicles [20, 45, 46]. Interestingly, GPC5-containing vesicle-like dots that have yet to start budding were also observed beneath the cell surface (S2F Fig). This image seems to reveal an intermediate process in the budding pathway, suggesting that the budded vesicles are generated from GPC5-Rab11-containing dots. Third, vesicles containing GPC5 and Rab11, ranging from 40 to 250 nm in diameter (Fig 8B), were released from U3DT cells. Taken together, our data strongly suggest that cell-surface GPC5 is transported in association with Rab11 inside cells.

Various organelles and components participate in cytokinesis, but it remains to be elucidated how each component (or organelle) interacts with the others. Although we demonstrated that GPC5 is located at the intercellular bridge during cell division, we could not elucidate the role of GPC5 during cytokinesis. Dynamic blebbing was observed during cytokinesis in U3DT cells. Blebbing appeared at late anaphase and disappeared after cytokinesis was completed, and the two daughter cells entered interphase. GPC5 accumulated in blebs, and in some blebs the signal was more condensed at the leading edge. This may indicate that GPC5 is correlated with the initial forward movement in blebs, as observed at the leading edge of migrating cells. The role of blebbing during cytokinesis is unknown. One possible explanation is that polarized blebbing movement induces mechanical separation of the daughter cells [32]. Support for this speculation comes from evidence that blebs tend to form at poles, away from the cleavage furrow (S2E Fig). Blebbing is a dynamic and rapid amoeboid movement, and faster-separating cells exert a stronger pull on their intercellular bridge [47].

Bleb growth decreased upon treatment of U3DT cells with RAB11A-siRNA. One possible explanation for this is that membrane sources for blebbing are diminished upon Rab11 inhibition. Bleb inflation is the result of unfolding of stored membrane, which could originate from fusion of Rab11 vesicles with plasma membrane [48, 49]. Alternatively, Rab11 inhibition may prevent cytoplasmic flow from pushing the membrane outwards.

GPC1-containing endosomes in the serum are a potential marker for early pancreatic cancer [33]. In this study, we detected EVs containing GPC5 in the conditioned medium of U3DT cells. These EVs were a heterogeneous mixture, varying in size from 40 to 250 nm in diameter. Moreover, we confirmed by immunostaining that GPC5-containing EVs associate with the exosome/microvesicle markers Rab11 and ARF6. Rab11 is required for exosome secretion, microvesicle budding, and viral budding [45, 46]. However, our results show that Rab11 not only promotes EV-budding, but is budded along with GPC5 into the EVs themselves. How GPC5 and Rab11 are recruited into EVs remains unknown.

Accumulating evidence indicate that EVs generated by highly aggressive cancer cells are capable of promoting tumor growth [50, 51]. We showed in Fig 9 that GPC5-containing EVs of U3DT cells are taken up by UE6E7T-3 cells, which GPC5 is expressed at low levels. Although GPC5-containing EVs emerged as an important player in U3DT cell proliferation, their effects on cell growth remain to be ascertained. Further work with purified GPC5-containing EVs might help to elucidate these effects.

**Fig 11:**
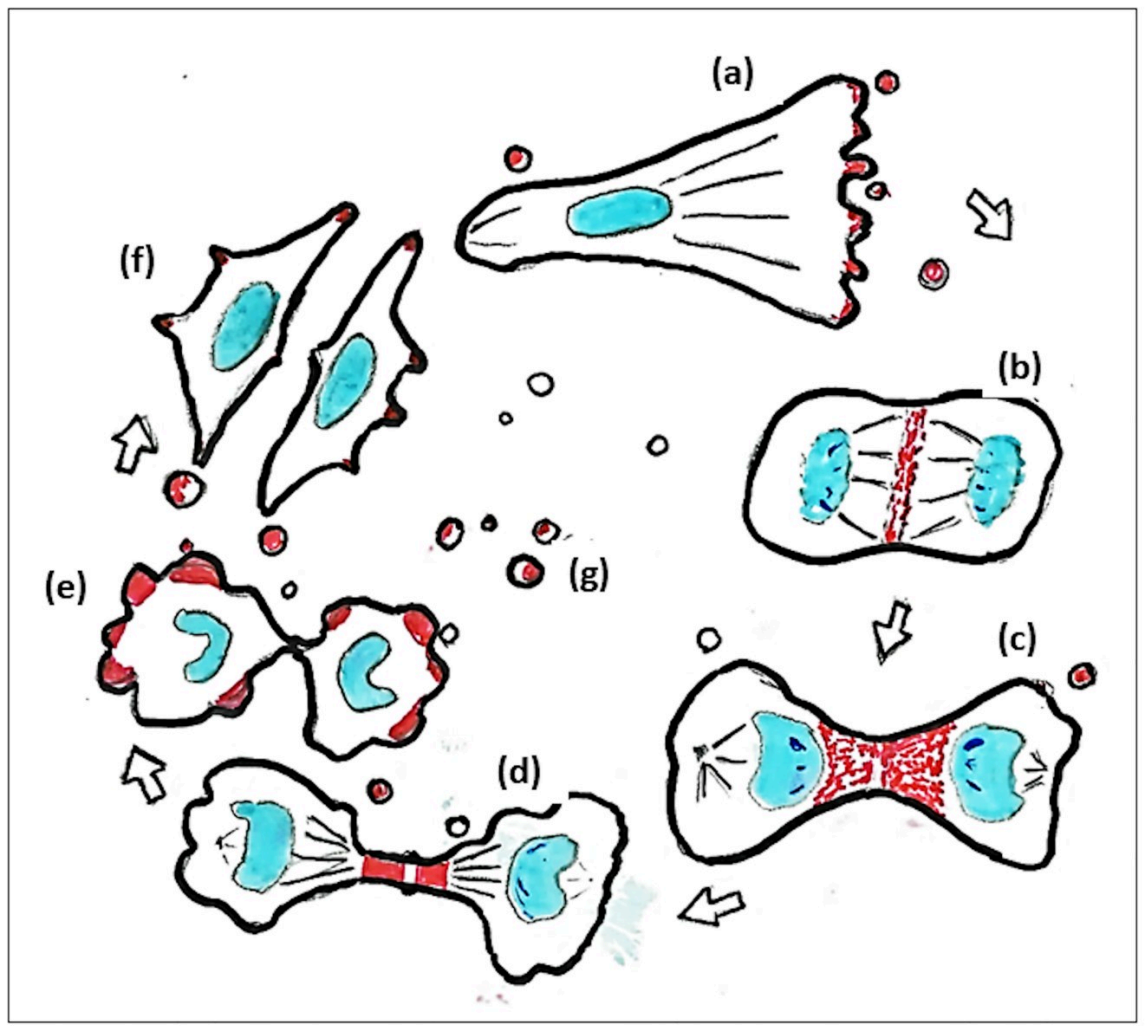
Summary. Subcellular localization of GPC5 during the cell cycle. (a) GPC5 (red) localizes at the leading edge of migrating cells, (b) at the equatorial plane, (c) at the furrow, (d) at the intercellular bridge during mitosis, (e) in membrane blebs during cytokinesis, (f) at the tips of filopodia, and (g) in EVs.

Previously, we showed that U3DT cells transformed after prolonged culture express significantly elevated levels of GPC5, localized to the plasma membrane. In this study, we showed that GPC5 also accumulates at the leading edges of migrating cells, intercellular bridges, and membrane blebs during cell division, and the EVs released from U3DT cells. These results are schematized in Fig 11. Multiple GPI-anchored proteins are internalized via dynamine-independent pathways, delivered to the Rab11-associated recycling endosomes, and recycled back to the plasma membrane [52]. The subcellular colocalization of GPC5, a GPI-anchored protein, with Rab11 or ARF6 raises the possibility that GPC5 is delivered via the same pathway as Rab11-associated recycling endosomes. Although the subcellular localization of GPC5 might be crucial for its functions, each function in turn might also play important role in promoting tumorigenesis by U3DT cells. Williamson et al. showed that GPC5 stimulates the proliferation of RMS cells [4]. Thus, the evidence obtained in this study should facilitate assessment of the functional contribution of GPC5-promoting proliferation in sarcoma, as well as the usefulness of EV as a biomarker for sarcoma.

## Conclusions

GPC5 localized not only to primary cilia on cell surface, but also to the leading edge of migrating cells, the intercellular bridge, and blebs during cytokinesis, and EVs. These localizations suggest that GPC5 play an important role in cell migration, which was abrogated by knockdown of GPC5. These findings might provide hints for assessment of the functional contribution of GPC5 to cell proliferation (Fig 11).

## Supporting information

S1 Fig Control staining for immunofluorescence. (A) UE6E7T-3 or U3DT cells were stained with goat anti-rabbit IgG (Alexa Fluor 488) and donkey anti-mouse IgG (Alexa Fluor 568). (B) As a negative control of GPC5, UE6E7T-3 cells were co-stained with anti-FGFR1 and anti-GPC5 antibodies. (C) U3DT cells were co-stained with WGA-Alexa Fluor 488 and with or without an anti-GPC5 mouse antibody.
(TIF)

S2 Fig Blebs in telophase cells. (A, C, E) Many blebs are observed outside two daughter cells. Telophase U3DT cell were stained with an anti-GPC5 antibody (red), Alexa Fluor 488-WGA (gray), an anti-Rab11A antibodies (green), and DAPI (blue). The immunostained cells were observed with a Leica SP8 confocal microscope. Scale bar: (A, C, E), 5 μm. (B, D, F). Blebs containing GPC5 and Rab11 are observed in telophase cells. Telophase U3DT cells were stained with an anti-GPC5 antibody (red), Alexa Fluor 488-WGA (gray), an anti-Rab11A antibodies (green), and DAPI (blue). Scale bar; (B, D, F), 2 μm.
(TIF)

S3 Fig Surface localization of GPC5 in U3DT cells treated with RAB11A-siRNA. Immunofluorescence images of trypsinized cells were obtained using a Leica SP-8 confocal microscope. Images of GPC5 (red), Rab11 (green), and DAPI (blue) in U3DT cells not treated (left) or treated (right) with RAB11A-siRNA. Scale bar, 5 μm.
(TIF)

S4 Fig Immunofluorescence images of FGFR1, Arf6, and Rab11 in U3DT cells treated with GPC5-siRNA (KD), non-targeting RNA (NT), or without siRNA (mock) for 1 day. (A) U3DT cells were co-stained with anti-GPC5 (red) and anti-FGFR1 (green) antibodies. (B) U3DT cells were co-stained with anti-GPC5 (red) and anti-ARF6 (green) antibodies. (C) U3DT cells were co-stained with anti-GPC5 (red) and anti-Rab11 (green) antibodies. Scale bars, 5 μm.
(TIF)
