# Supplementary figures and images for: Subcellular localization of glypican-5 is associated with dynamic motility of the human mesenchymal stem cell line U3DT
Source: PLoS One. 2021 Feb 19;16(2):e0226538. doi: 10.1371/journal.pone.0226538 (PMC7895401; doi:10.1371/journal.pone.0226538)

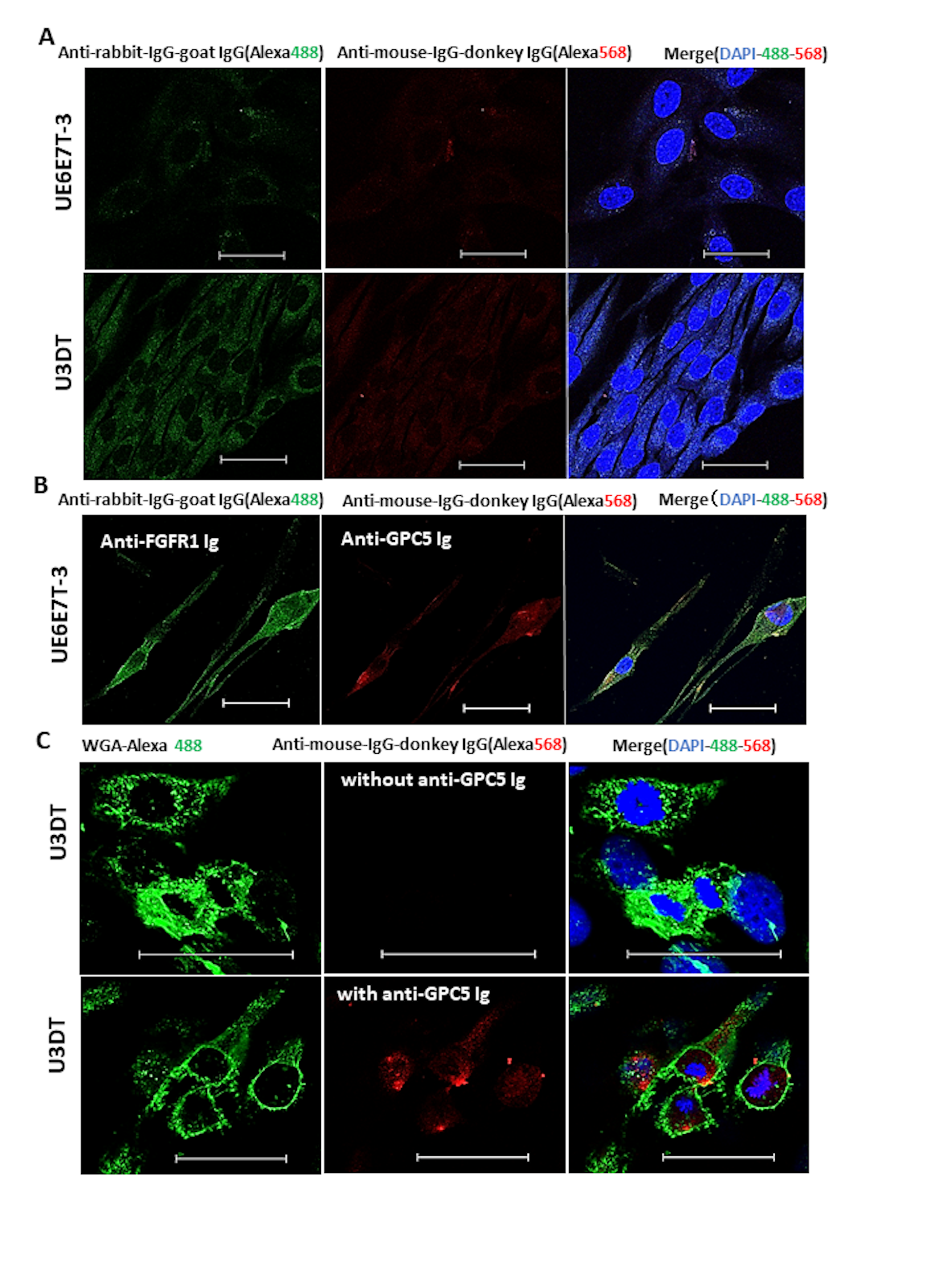

Supplement: S1 Fig — (A) UE6E7T-3 or U3DT cells were stained with goat anti-rabbit IgG (Alexa Fluor 488) and donkey anti-mouse IgG (Alexa Fluor 568). (B) As a negative control of GPC5, UE6E7T-3 cells were co-stained with anti-FGFR1 and anti-GPC5 antibodies. (C) U3DT cells were co-stained with WGA-Alexa Fluor 488 and with or without an anti-GPC5 mouse antibody. (TIF) [file pone.0226538.s001.tif]

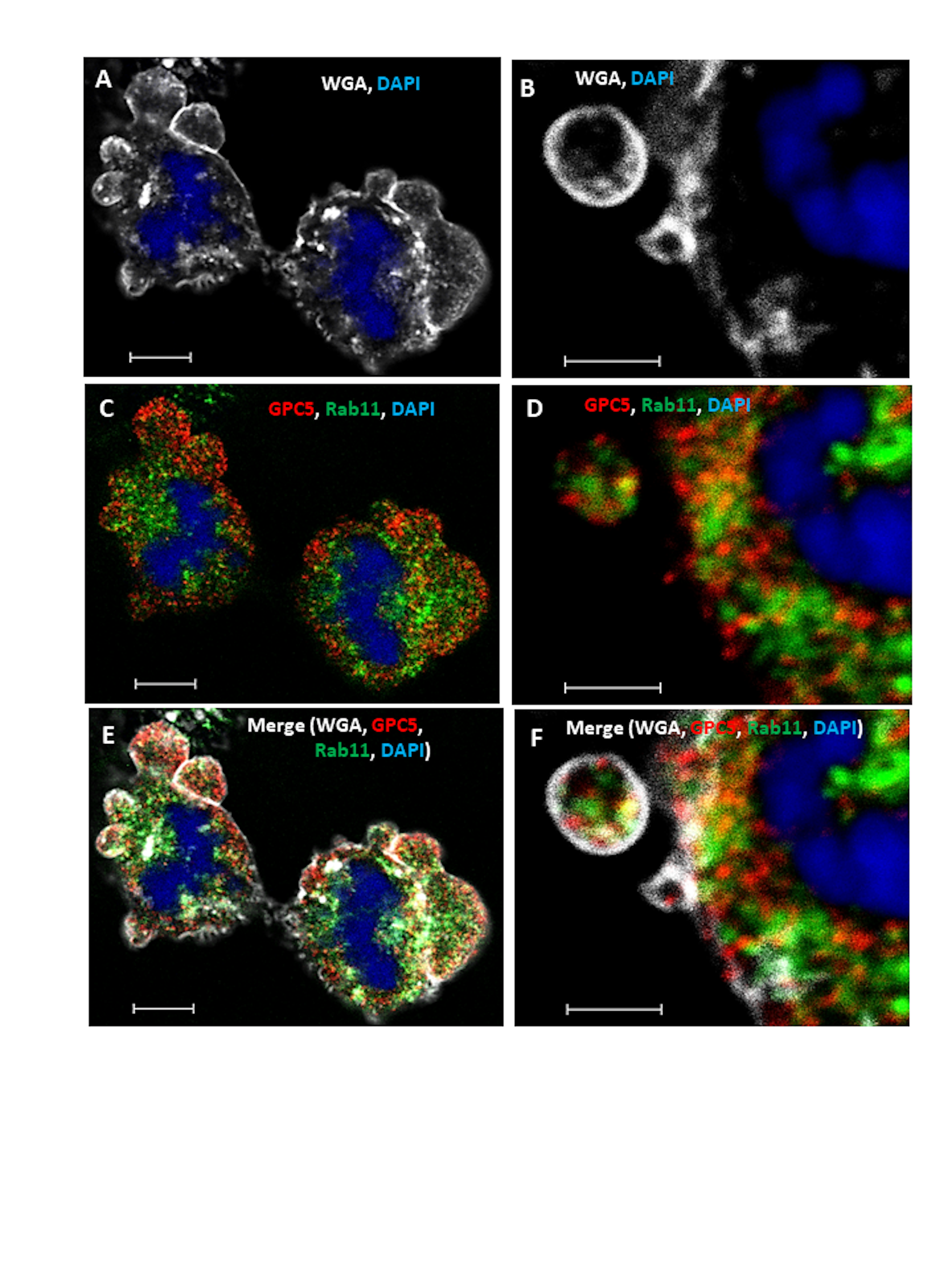

Supplement: S2 Fig — (A, C, E) Many blebs are observed outside two daughter cells. Telophase U3DT cell were stained with an anti-GPC5 antibody (red), Alexa Fluor 488-WGA (gray), an anti-Rab11A antibodies (green), and DAPI (blue). The immunostained cells were observed with a Leica SP8 confocal microscope. Scale bar: (A, C, E), 5 μm. (B, D, F). Blebs containing GPC5 and Rab11 are observed in telophase cells. Telophase U3DT cells were stained with an anti-GPC5 antibody (red), Alexa Fluor 488-WGA (gray), an anti-Rab11A antibodies (green), and DAPI (blue). Scale bar; (B, D, F), 2 μm. (TIF) [file pone.0226538.s002.tif]

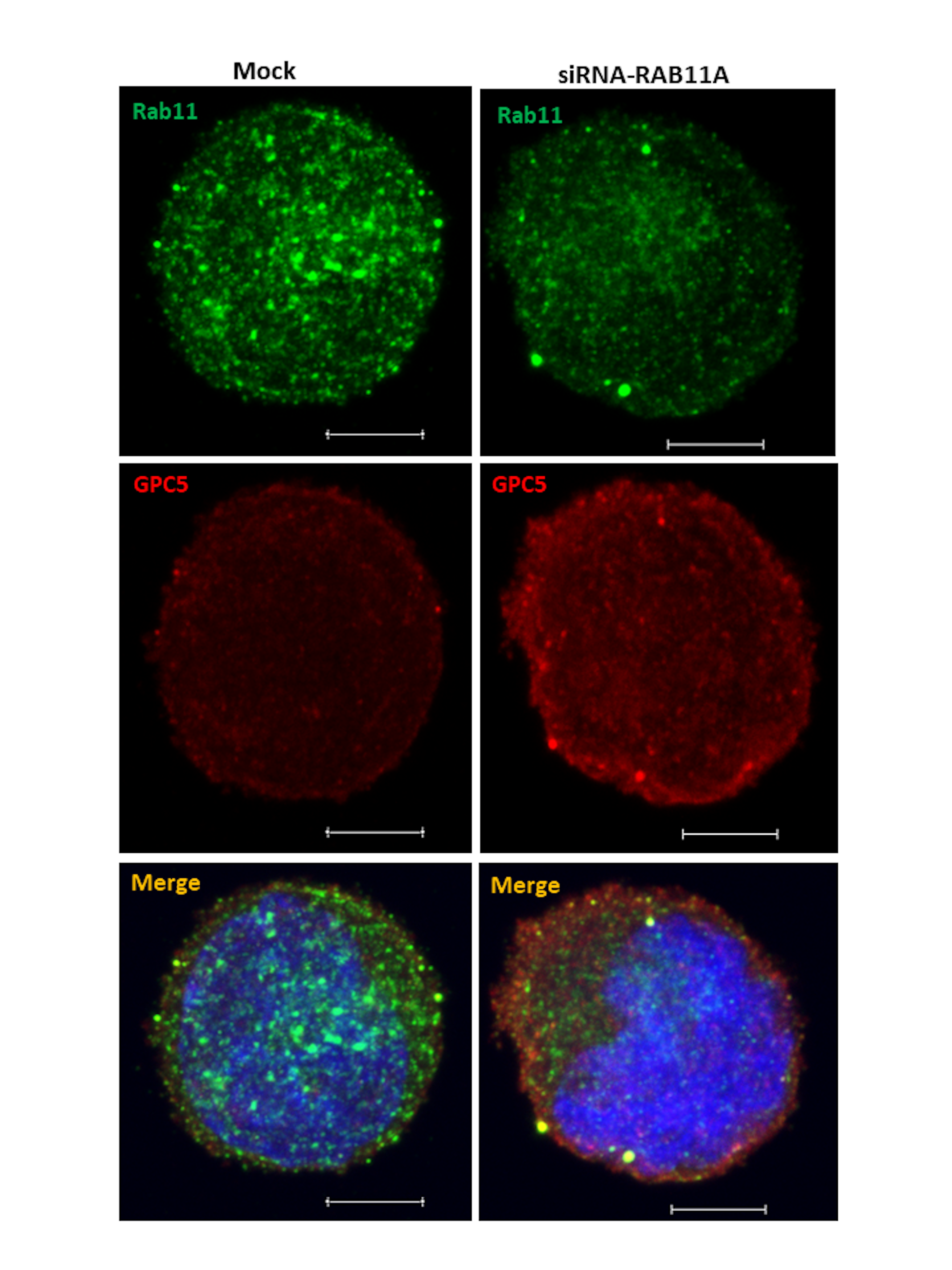

Supplement: S3 Fig — Immunofluorescence images of trypsinized cells were obtained using a Leica SP-8 confocal microscope. Images of GPC5 (red), Rab11 (green), and DAPI (blue) in U3DT cells not treated (left) or treated (right) with RAB11A-siRNA. Scale bar, 5 μm. (TIF) [file pone.0226538.s003.tif]

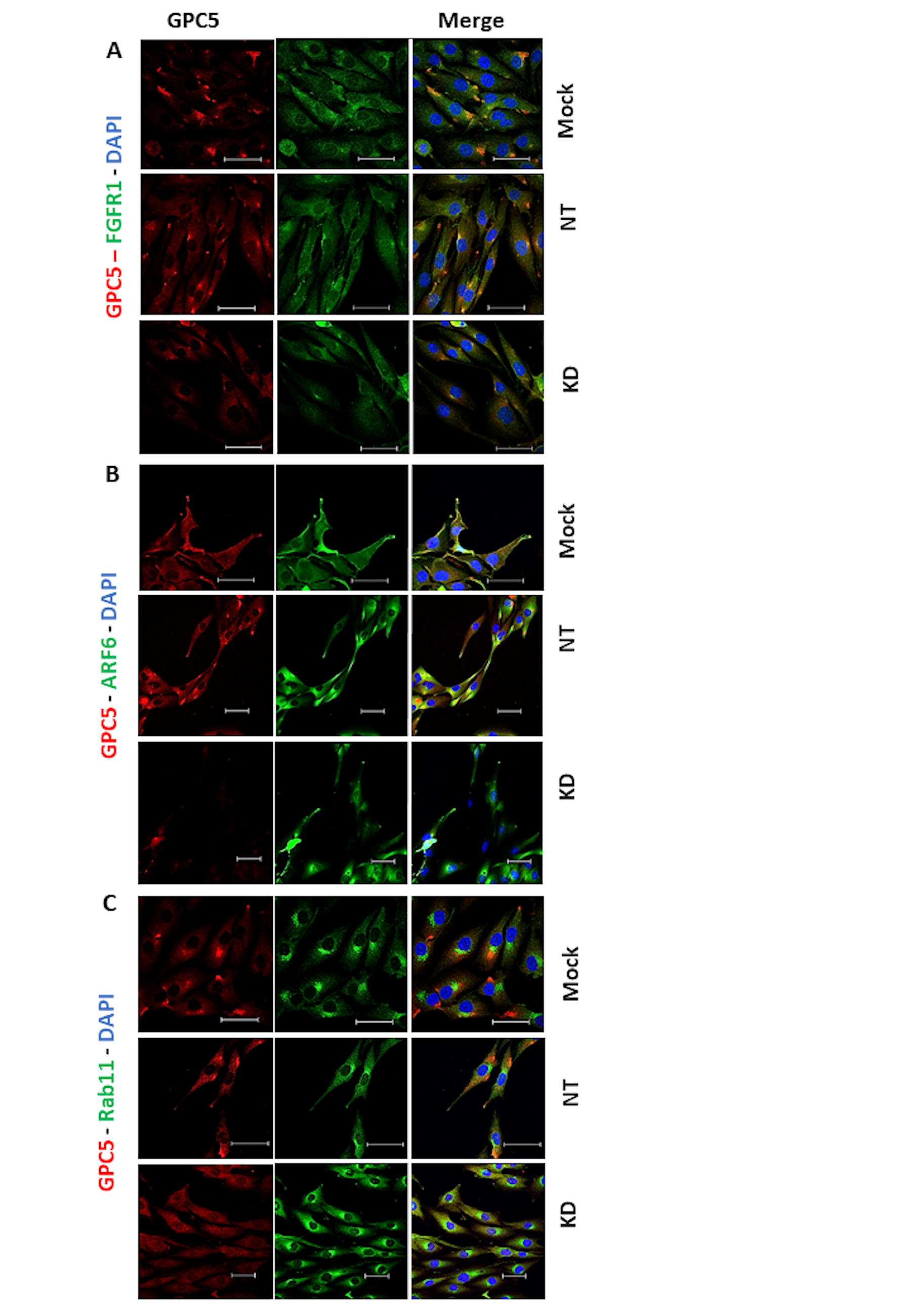

Supplement: S4 Fig — (A) U3DT cells were co-stained with anti-GPC5 (red) and anti-FGFR1 (green) antibodies. (B) U3DT cells were co-stained with anti-GPC5 (red) and anti-ARF6 (green) antibodies. (C) U3DT cells were co-stained with anti-GPC5 (red) and anti-Rab11 (green) antibodies. Scale bars, 5 μm. (TIF) [file pone.0226538.s004.tif]
